# Supplementary material for: Increasing coverage in cervical and colorectal cancer screening by leveraging attendance at breast cancer screening: A cluster-randomised, crossover trial
Source: PLoS Med. 2024 Aug 13;21(8):e1004431. doi: 10.1371/journal.pmed.1004431 (PMC11321549; doi:10.1371/journal.pmed.1004431)
Supplement: S1 Questionnaire — (DOCX) [file pmed.1004431.s001.docx]

**Questionnaire**

Questions in the questionnaire, freely translated from Danish by the first author.

**Satisfaction with breast cancer screening:**Questionnaire sent to women in both the intervention and the control group

Q1 Feeling welcome:

How was your experience with the reception, when you arrived at the screening unit?

1. Very good
2. Good
3. Poor
4. Really poor
5. Don’t know

Q2 Professionalism:

Did the staff act professionally at the mammogram?

1. To a great extent
2. To a certain extent
3. To a lesser extent
4. Not at all
5. Don’t know

Q3 Trust in examination:

Did you trust the way the staff executed the examination?

1. To a great extent
2. To a certain extent
3. To a lesser extent
4. Not at all
5. Don’t know

Q4 Overall satisfaction:

Overall, are you satisfied with your visit in the breast cancer screening unit?

1. To a great extent
2. To a certain extent
3. To a lesser extent
4. Not at all
5. Don’t know

Q5 Intention to participate next time:

Do you intend to participate when you receive the next invitation to breast cancer screening?

1. Yes
2. No
3. Don’t know
4. Not relevant

**Evaluation of the intervention:**

Questionnaire sent to women in the intervention group

Q1 Oral information:
Was the oral information about the project understandable?

1. To a great extent
2. To a certain extent
3. To a lesser extent
4. Not at all
5. Don’t know

Q2 Written information:
Was the written information about the project understandable?

1. To a great extent
2. To a certain extent
3. To a lesser extent
4. Not at all
5. Don’t know

Q3 Sufficient information:
Did you receive all the information you needed?

1. To a great extent
2. To a certain extent
3. To a lesser extent
4. Not at all
5. Don’t know

Q4 Meaningfulness:

Was it meaningful for you to be asked questions about your participation in the other screening programmes?

1. To a great extent
2. To a certain extent
3. To a lesser extent
4. Not at all
5. Don’t know

Q5 Combined screening:
Do you think it would be a good opportunity to combine all three cancer screenings at once?

1. To a great extent
2. To a certain extent
3. To a lesser extent
4. Not at all
5. Don’t know

Q6 Would participate another time:

If you were offered to have a check-up on your cervical and colorectal cancer screening status another time, would you accept the offer?

1. Yes
2. No
3. Don’t know
