# Supplementary material for: Increasing coverage in cervical and colorectal cancer screening by leveraging attendance at breast cancer screening: A cluster-randomised, crossover trial
Source: PLoS Med. 2024 Aug 13;21(8):e1004431. doi: 10.1371/journal.pmed.1004431 (PMC11321549; doi:10.1371/journal.pmed.1004431)
Supplement: S1 Table — (DOCX) [file pmed.1004431.s003.docx]

**Sensitivity analysis of primary outcomes**

**S1 Table.** Differences in coverage and participation between intervention and control groups in cervical and colorectal cancer screening accounting for clustering within screening units.

|  | Risk difference  (Percentage points)  [95% CI] |
| --- | --- |
| Cervical cancer screening | |
| Coverage at baseline | 0.6  [-1.4, 2.5] |
| Coverage six months after intervention | 4.8  [3.6, 5.9] |
| Screening participation for women overdue at baseline | 25.8  [19.3, 32.4] |
| Colorectal cancer screening | |
| Coverage at baseline | -0.2  [-2.3, 1.9] |
| Coverage six months after intervention | 3.8  [1.8, 5.8] |
| Screening participation for women overdue at baseline | 14.9  [10.1, 19.8] |

CI, confidence interval
