# Supplementary material for: Increasing coverage in cervical and colorectal cancer screening by leveraging attendance at breast cancer screening: A cluster-randomised, crossover trial
Source: PLoS Med. 2024 Aug 13;21(8):e1004431. doi: 10.1371/journal.pmed.1004431 (PMC11321549; doi:10.1371/journal.pmed.1004431)
Supplement: S2 Table — (DOCX) [file pmed.1004431.s004.docx]

**Descriptive questionnaire data**

**S2 Table.** Satisfaction with breast cancer screening (in the total study population, N=17,059)

|  | **1**  **(Most positive)**  n (%) | | **2**  n (%) | | **3**  n (%) | | **4**  **(Most negative)**  n (%) | | **“Do not know”** | |
| --- | --- | --- | --- | --- | --- | --- | --- | --- | --- | --- |
|  | **I** | **C** | **I** | **C** | **I** | **C** | **I** | **C** | **I** | **C** |
| **Q1. Feeling welcome** | 1,895 (51.4%) | 6,874 (51.4%) | 1,710 (46.4%) | 6,116 (45.7%) | 43 (1.2%) | 218 (1.6%) | 4 (0.1%) | 47 (0.4%) | 36 (1.0%) | 116 (0.9) |
| **Q2. Professionalism** | 3,408 (92.4%) | 12,234 (91.5%) | 230 (6.2%) | 955 (7.1%) | 36 (1.0%) | 118 (0.9%) | 4 (0.1%) | 30 (0.2%) | 10 (0.3%) | 34 (0.3%) |
| **Q3. Trust in examination** | 3,350 (90.8%) | 12,067 (90.3%) | 276 (7.5%) | 1,083 (8.1%) | 39 (1.1%) | 145 (1.1%) | 19 (0.5%) | 54 (0.4%) | 4 (0.1%) | 22 (0.2%) |
| **Q4. Overall satisfaction** | 3,357 (91.0%) | 12,015 (89.9%) | 285 (7.7%) | 1,121 (8.4%) | 34 (0.9%) | 178 (1.3%) | 12* (0.3%) | 57* (0.4%) |  |  |
| **Q5. Intention to participate next time** | **Yes** | | **No** | |  |  |  |  |  |  |
|  | **I** | **C** | **I** | **C** |  |  |  |  |  |  |
|  | 3,499 (99.7%) | 12,773 (99.6%) | 11 (0.3%) | 52 (0.4%) |  |  |  |  |  |  |

Abbreviations: I, Intervention group; C, Control group

Survey responses were provided on a Likert scale ranging from 1 to 4 or “Do not know”, except for Q5 where responses were “yes” or “no”. In Q5, women indicating that this was not relevant for them were excluded.

* Numbers have been aggregated in Q4 when responding with “4” or “Do not know” to comply with Danish data protection legislation, which restricts the reporting of small numbers.
