# Supplementary material for: Increasing coverage in cervical and colorectal cancer screening by leveraging attendance at breast cancer screening: A cluster-randomised, crossover trial
Source: PLoS Med. 2024 Aug 13;21(8):e1004431. doi: 10.1371/journal.pmed.1004431 (PMC11321549; doi:10.1371/journal.pmed.1004431)
Supplement: S3 Table — (DOCX) [file pmed.1004431.s005.docx]

**Descriptive questionnaire data**

**S3 Table.** Evaluation of the intervention (women in the total intervention group, who have responded to the questionnaire, N=3,554)

|  | **1**  **(Most positive)**  n (%) | **2**  n (%) | **3**  n (%) | **4**  **(Most negative)**  n (%) | **”Do not know”**  n (%) |
| --- | --- | --- | --- | --- | --- |
| **Q1. Oral information** | 3,299 (90.9%) | 247 (7.0%) | 26 (0.7%)* | | 52 (1.5%) |
| **Q2. Written information** | 2,808 (79.0%) | 339 (9.5%) | 13 (0.4%) | 9 (0.3%) | 385 (10.8%) |
| **Q3. Sufficient information** | 3,155 (88.8%) | 267 (7.5%) | 24 (0.7%) | 8 (0.2%) | 100 (2.8%) |
| **Q4. Meaningfulness** | 2,150 (60.5%) | 824 (23.2%) | 370 (10.4%) | 117 (3.3%) | 93 (2.6%) |
| **Q5. Combined screening** | 1,849 (52.0%) | 703 (19.8%) | 375 (10.6%) | 132 (3.7%) | 495 (13.9%) |
|  | **Yes** | **No** |  |  |  |
| **Q6. Would participate another time** | 2,994 (87.4%) | 162 (4.7%) |  |  | 270 (7.9%) |

Survey responses were provided on a Likert scale ranging from 1 to 4 or “Do not know”, except for Q6 where responses were “yes”, “no” or “Do not know”. In Q6, women indicating that this was not relevant for them were excluded.

* Numbers have been aggregated in Q1 when responding with “3” or “4” to comply with Danish data protection legislation, which restricts the reporting of small numbers.
