# Supplementary material for: Increasing coverage in cervical and colorectal cancer screening by leveraging attendance at breast cancer screening: A cluster-randomised, crossover trial
Source: PLoS Med. 2024 Aug 13;21(8):e1004431. doi: 10.1371/journal.pmed.1004431 (PMC11321549; doi:10.1371/journal.pmed.1004431)
Supplement: S4 Table — (DOCX) [file pmed.1004431.s006.docx]

**Descriptive questionnaire data**

**S4 Table.** Evaluation of the intervention (women in the intervention group eligible for both screening programmes*, who have responded to the questionnaire, N=2,063)

|  | **1**  **(Most positive)**  n (%) | **2**  n (%) | **3**  n (%) | **4**  **(Most negative)**  n (%) | **”Do not know”**  n (%) |
| --- | --- | --- | --- | --- | --- |
| **Q1. Oral information** | 1,865 (90.4%) | 154 (7.5%) | 15 (0.7%)** | | 29 (1.4%) |
| **Q2. Written information** | 1,602 (77.7%) | 209 (10.1%) | 6 (0.3%) | 5 (0.2%) | 241 (11.7%) |
| **Q3. Sufficient information** | 1,833 (88.9%) | 160 (7.8%) | 15 (0.7%) | 4 (0.2%) | 51 (2.5%) |
| **Q4. Meaningfulness** | 1,277 (61.9%) | 451 (21.9%) | 214 (10.4%) | 66 (3.2%) | 55 (2.7%) |
| **Q5. Combined screening** | 1,113 (54.0%) | 407 (19.7%) | 229 (11.1%) | 80 (3.9%) | 234 (11.3%) |
|  | **Yes** | **No** |  |  |  |
| **Q6. Would participate another time** | 1,775 (87.9%) | 89 (4.4%) |  |  | 156 (7.7%) |

Survey responses were provided on a Likert scale ranging from 1 to 4 or “Do not know”, except for Q6 where responses were “yes”, “no” or “Do not know”. In Q6, women indicating that this was not relevant for them were excluded.

* Women eligible for both cervical cancer screening and colorectal cancer screening (aged 50-64 years, no prior cervical or colorectal cancer diagnosis, no hysterectomy and with more than 4.5 months remaining to respond to the first invitation in colorectal cancer screening if aged 50 years).

** Numbers have been aggregated in Q1 when responding with “3” or “4” to comply with Danish data protection legislation, which restricts the reporting of small numbers
