# Supplementary material for: Increasing coverage in cervical and colorectal cancer screening by leveraging attendance at breast cancer screening: A cluster-randomised, crossover trial
Source: PLoS Med. 2024 Aug 13;21(8):e1004431. doi: 10.1371/journal.pmed.1004431 (PMC11321549; doi:10.1371/journal.pmed.1004431)
Supplement: Attachment — Submitted filename: Response to Reviewers_020524.docx [file pmed.1004431.s007.docx]

Dear Alexandra Schaefer

Thank you for giving us the opportunity to revise our manuscript.

Below, is our detailed response to the reviewer’s comments, presented point by point. Sentences beginning with numbers are copies of the reviewers’ comments; our answers are in italics. The comments have been addressed comprehensively, and we appreciate the valuable feedback provided by the reviewers.

We hope that our revisions meet your expectations. Please do not hesitate to reach out in case of clarifying questions. We look forward to your feedback.

Yours sincerely,

Anne Dorte Helgestad

*On behalf of all authors*

Editor: We agree with the reviewers that more details should be provided on screening rates and screening programs in Denmark. We also ask you to discuss the potential for selection bias, as individuals who attend screening for one reason are more likely to accept screening.

*Thank you for your comments. We have revised the introduction section, now including a brief introduction to the screening programmes before providing a detailed description in the methods section. Additionally, we have included a paragraph in the discussion addressing the risk of selection bias. We hope these revisions meet your expectations*

Reviewer #1: excellent project. excellent methods and analysis.

Reviewer #2: The authors present results from an elegant cluster randomised crossover trial of offering three cancer screening tests at one clinical setting to test whether it improves uptake and overall coverage.

The title of the manuscript uses common parlance of "three birds with one stone" which I find unacceptable in a scientific report. The authors must replace this with a more suitable medically descriptive title. In addition I have noted further issues as detailed below.

*Response: We have changed the title accordingly.*

Issues identified:

1.	Authors state no funding received for this work but then mention under competing interests that the cobas 4800 HPV tests were sponsored by Roche. Is this not funding? I find it hard to believe there was no specific funding allocated for the trial. Was there not institute funding for the staff and /or student time for conducting the trial?

Response:

The project was embedded in the CDR’s breast cancer screening programme and did not receive any external funding.

In the manuscript, we have revised the abstract:

*“Roche Diagnostics sponsored HPV test kits for cervical cancer screening; no other external funding was received. “*

2.	There are a few English readability errors in the abstract, such as on line 29: "Screening participation (in Denmark) remain(s) suboptimal in …."

*Response: Thank you for bringing this to our attention. We have carefully revised the manuscript to address errors and improve the readability.*

3.	It would be more correct to state "total screening coverage for the three programmes" than "overall coverage" in the abstract, as well as in the body of the text.

*Response: We have reviewed the manuscript and revised the wording of “overall coverage” to “total coverage” as recommended by the reviewer.*

4.	The introduction, from lines 63 to 73 makes the case that the main reason for non-participation in screening is "temporary impediments or forgetfulness". The discussion lines 436-437 also speaks of these "individual barriers". In my experience of cancer screening programme research, this is an impressively large oversimplification. It is a significant weakness of this study that it does not consider equity, access, and socioeconomic factors as barriers to screening. These factors are raised by the authors, for the first time, in the discussion on lines 449 to 461, although not as an admission of a study weakness which must be addressed.

*Response: Thank you for addressing this and for the opportunity to enhance the manuscript within these aspects. We completely agree that there are numerous reasons for non-participation, and our study only address some of these barriers.*

*We have revised the sentence in the introduction (lines 95-96) to emphasize that various barriers exist, and that we only discuss a few:*

*“Among numerous barriers, one reason mentioned across screening programmes is temporary impediments or forgetfulness.”*

*And elaborated on this in the discussion (lines 504-507):*

*“The intervention tackles several barriers commonly associated with non-participation, including information, support, screening accessibility and logistical challenges. However, other barriers, such as cultural factors, language barriers, attitudes towards screening and socioeconomic status, were not directly addressed by the intervention.”*

5.	Outcomes, line 166 onward: Terminology could be improved. Difference between the control and intervention group with respect to CC and or CRC screening (uptake) six months after the (offer of self-testing) for women who were overdue for CC/CRC screening at the intervention date.

*Response: We acknowledge the complicated terminology in our composite outcome measures including both CC and CRC screening and the need for clarity in describing these measures.*

*We have revised the terminology and hope the revisions we have made address your concerns.*

*“1) Difference in total CC and/or CRC screening coverage between the intervention and control groups six months after the intervention. Assessed as the proportion of women adhering to CC screening for the past 3.5/5.5 years (depending on the woman’s age) and/or adhering to CRC screening for the past two years and 4.5 months.*

*2) Difference in CC and/or CRC screening participation six months after the intervention between the intervention and control groups among women overdue for CC/CRC screening at the intervention date.”*

6.	In your methods:

a.	You need a section in the methods briefly describing the ethics approval for the study and the process of informed consent undertaken for participants.

*Response: We have revised the Ethics Statement (lines 296-300) with the following:*

*“The study was listed in the record of processing activities for research projects in the Central Denmark Region (R. No.: 1-16-02-217-21). In pursuance of the Danish Consolidation Act on Research Ethics Review of Health Research Projects, the study was not notifiable to the Committee (R. No.: 1-10-72-1-21) and informed consent was therefore not required.”*

b.	It is not clear that HPV self sampling is not already implemented in your programme, this is only mentioned in the discussion in lines 426-427. There is also no information on the method of your self sampling test (cobas 4800), whether it is validated for self sampling in your setting and the self sampling device used. Please update your methods with a section on all this information.

*Response: Thank you for the opportunity to elaborate on our methods.*

*In line 139 in the methods, the lack of a self-sampling offer in the study period is mentioned. In the intervention section, lines 179-182, the self-sampling devices offered are mentioned.*

*To provide more clarity, we have revised the methods section and added a section with an elaborated version of the methods (line 201):*

*“****Follow-up and clinical management***

*Follow-up and clinical management were embedded into the Danish routine cancer screening programmes and national guidelines were followed. Consequently, vaginal self-samples were analysed for high-risk HPV (HPV16, HPV18 and 12 other high-risk types pooled together; HPV 31, 33, 35, 39, 45, 51, 52, 56, 58, 59, 66, 68) using the Cobas 4800 HPV DNA test (1). The analyses were conducted as part of routine and validated laboratory procedures at the Department of Pathology at Randers Regional Hospital (2). Women with an HPV-positive self-sampled screening were advised to schedule an appointment with their GP within one month for cervical cytology sample collection for triage purposes (3). The triage process involved co-testing for HPV and cytology, which guided further clinical follow-up (4). FIT samples were analysed for the presence of blood traces using a cut-off value of 100ng haemoglobin (HB)/mL buffer at the Department of Clinical Biochemistry at Randers Regional Hospital, following national protocols (5). Women with a positive FIT were booked for a colonoscopy within 14 days, and further clinical management was in accordance with national guidelines.”*

c.	It is not clear until the discussion that the self test kits were mailed out to participants, after the breast screening accession, and that women weren't able to do the test onsite.

*Response: In the methods section, lines 177-184, we have provided a description of the intervention, which includes the process of mailing self-sampling kits after the intervention. We have revised the paragraph to enhance clarity of the process.*

*“Women eligible for CC screening but overdue for screening were offered to receive a vaginal brush (Evalyn Brush, Rovers Medical Devices, Netherlands (6)) for HPV self-sampling. Women overdue for CRC screening were offered to receive a FIT (OC Sensor System, Eiken Chemical Company, Japan) for blood trace detection. Both screening kits were forwarded by mail after the intervention date along with screening programme information and picture-based information material showing how to use the self-sampling kit.”*

d.	Method: how are participants managed clinically? Do you not have an algorithm for directing those who are HPV16/18 straight to colposcopy? With those positive for non-16/18 HPV types given cytology and triage? Brief information on this would be helpful.

*Response: All women who tested positive for HPV through self-sampled screening were notified via mail and advised to schedule an appointment with their GP within 30 days to have a cervical cytology sample taken for triage, irrespective of HPV type detected at the self-sample. This approach aligns with national guidelines for the clinical management of HPV self-samples. Subsequent clinical management is determined by the results of the GP-collected sample, which includes co-testing for both HPV types and cytology, as well as the woman’s age.*

*In the revised version this is described in the ‘Follow-up and clinical management’ section, starting from line 201 and onward.*

e.	Method: Study exclusion criteria, did you not have a category for loss to follow-up due to non-contactability? Or was this not an issue?

*Response: In this study, non-contactability did not emerge as an issue for loss to follow-up. All follow-up data collected at the end of the six-months period were extracted from registers.*

7.	Results:

a.	Table 5 the section on "satisfaction with breast cancer screening" it is not clear what this data relates to. Is it the 1-2 positive responses on the Likert scale, as for the "Evaluation of the intervention"? Please correct the legend associated with this figure

*Response: Thank you for bringing the error to our attention. We have corrected the legend as follows:*

*“*Survey responses were given on a Likert scale ranging from 1 to 4. The reported values are the frequency and percentage of "1-2" (positive) Chi-squared tests compare "1-2" (positive) to "3-4" (negative).”*

b.	Table 4. Was there any statistical difference between the groups? I.e. in the CC groups, was there any difference between uptake in the unscreened and underscreened groups? Similarly for the CRC groups, was there any difference between unscreened and underscreened groups?

*Response:* *Thank you for your inquiry. Following revision, we have included this information to the results sections, lines 338 and 348:*

*CC screening:*

*“Unscreened women participated statistically significantly more than under-screened women (p=0.02).”*

*CRC screening:*

*“No statistically significant difference in participation was found between under-screened and unscreened women (p=0.81).”*

c.	Table 2. I note that some columns do not add up to the total N due to missing data, I would prefer that you add a line to the ethnicity, marital status, and education rows for "data not given" to enable easy viewing of the impact of this on the results. By my calculations, it ranges from 4 for the CC intervention groups to 146 in the control group for education.

*Response: Concerning missing data, only descriptive data for sociodemographic variables were absent for a very small proportion of the study population. Due to Danish data protection Legislation, we are restricted from presenting specific numbers due to their limited size.*

8.	Discussion: In addition to addressing the weakness of your study I addressed in point 4, the statement and reference on line 460 that "offering self-sampling at GPs has shown promising results" should be removed as it is is out of date. Self sampling for Cervical cancer screening is now implemented in GP clinics, via mailout and many other settings in many countries, including the Netherlands (since 2017), Australia and New Zealand. Indeed, the metaanalysis by Costa et al (2023) that is cited in the discussion, found that "overall screening participation was higher among women invited for self-sampling compared to control, regardless of the invitation strategy used."

*Response: Thank you for the comment. We acknowledge the varying pace of implementation of self-sampling offers across different countries. In response to your feedback, we have revised the text and omitted the sentence.*

Reviewer #3: Statistical review

This paper reports a cluster crossover trial that evaluates whether an intervention is successful at improving screening for cervical and colorectal cancer screening. The design used is very interesting and appears to be very efficient, made possible by well-linked routinely collected data.

I had some comments on the statistical methods and reporting below.

9.	Abstract: I would briefly add how the data was analysed.

10.	Abstract: I would use 'primary outcomes' rather than 'main outcomes'

11.	Abstract: I would provide a p-value for the primary endpoints (presumably each will be p<0.001). I will leave it to the authors whether to include it for the subgroup of overdue women

*Response: We appreciate the suggestions, and we have accordingly revised the abstract.*

12.	Page 7: I wouldn't describe death/emigration as an exclusion criterion as it's post baseline - I would include this in the definition of an analysis population (which might be modified intention to treat).

*Response: Thank you for the comment. We have revised the text and figures accordingly (lines 158-160):*

“*The exclusion criteria included a prior diagnosis of CC/CRC and prior hysterectomy (only in the CC screening). Women who were lost to follow-up (death or emigration within six months after the intervention) were not included in the analyses.”*

13.	Page 7 - was the random allocation simple randomisation or were there restrictions (e.g. it sounds like towards the end of the paragraph that it might have involved block randomisation with one block).

*Response: As stated in the beginning of the paragraph, the study was designed as a cluster-randomised, crossover trial conducted with 100 cluster periods across the five BC screening units located in the CDR. During each intervention day, one unit served as intervention cluster while the remaining four units served as control clusters. Hence, it was neither a simple randomisation nor a conventional cluster randomization, as each unit had an equal number of days as both intervention and control clusters. This approach was implemented to ensure that population characteristics associated with each unit would not introduce systematic differences between the intervention and control groups.*

*The following has been added to clarify (lines 169-171):*

*“Accordingly, all BC screening units had 20 days of intervention and 80 days serving as controls. This approach was adopted to avoid the potential introduction of systematic differences between the intervention and control groups due to variations in the populations associated with each screening unit.”*

14.	Page 10 - I notice the clinicaltrials.gov registration page contains quite a few secondary outcomes: I'd recommend these are acknowledged and either reported in this paper or a statement added that the secondary outcomes would be reported in another publications. Some are reported in the results (although not mentioned in the methods) and some don't seem to be (e.g. incidence of CIN2+).

*Response: Thank you for the comment. In the methods section, we have elaborated on this matter with the following (lines 230-235):*

*“In pursuance of Danish data protection legislation, further clinical follow-up, as outlined in the protocol (histology after biopsies obtained at colposcopies/colonoscopies and referral rate for colposcopy after an HPV-positive CC screening), is not reported due to the small numbers of cases.*

*Secondary outcomes also included the proportion of women eligible for all three programmes, the proportion of these women overdue for both CC and CRC screening, and their participation in both screenings within the follow-up period.”*

15.	Page 12 - can more details about how the cluster crossover trial design was taken into account with the sample size calculation? E.g. was any correlation within clusters considered? I note later that it wasn't, so perhaps a statement saying this should be added.

*Response: We have added this on lines 276-278:*

*“In this calculation, the potential effect of the cluster-randomised, crossover design was not considered, as the clusters served equally as intervention and control groups.”*

16.	Page 12 - I was not convinced by the argument to not account for clustering in the analysis - it is possible that individuals within clusters are more similar in outcomes than individuals outside the cluster. I would therefore recommend that a secondary analysis allows for clustering, e.g. through having a random effects logistic regression model with random effect for site included.

*Response: Since each of the breast cancer screening units served an equal number of times as intervention and control units, there should be no difference between the intervention group and the control group.*

*However, as recommended, we have added a secondary analysis in the supplements demonstrating that there was no effect of the cluster-randomised, crossover design. The following has been added in the methods section (287-289):*

*"Hence, a design effect due to cluster randomisation was not considered in the main analyses. However, a sensitivity analysis of the primary outcomes was conducted using logistic regression with cluster-robust standard errors to account for clustering within screening units."*

*And the following to the results section (lines 250-251):*

*“The sensitivity analysis of the primary outcomes, accounting for clustering within the screening units, did not alter the estimates (Supplemental material).”*

17.	Table 2 - I am not convinced that including p-values in a baseline table is needed with this study design. Due to the random selection any significant findings would be known to be type I errors.

*Response: Thank you for your feedback. We appreciate your point regarding the inclusion of p-values in the baseline table of a randomised trial, and acknowledge that any observed differences could be attributed to chance. Taken your suggestions into consideration, we have removed the p-values from the table accordingly. We appreciate your thorough review and the opportunity to enhance the quality of our manuscript.*

18.	Table 3 - 4 -as per above comments I would add p-values for the outcome results (but not baseline results).

*Response: Thank you for the suggestion, which we have now implemented to enhance the transparency of the results.*

19.	Table 5 - the rightmost column is presumably the p-value from a chi-squared test rather than chi-squared test itself.

*Response: Thank you for bringing the error to our attention. We have now corrected the column text*.

Reviewer #4: This article focuses on screening for breast and cervical cancer, two of the most prevalent cancers that, if caught earlier, can lead to positive health outcomes. Overall, the study approach appears to be sound. The minor recommendations offered below seek to further enhance the paper’s readability and impact in the greater cancer screening literature.

Introduction

20.	-As this is an international journal, it would be beneficial to, in the introduction, very briefly provide additional information about BC and CC rates and screening programs in Denmark (e.g. what is the age eligibility criteria, are screening reminders sent directly to eligible individuals or done via physician, is screening free?/reduced cost?). This would set the reader up for the more in-depth overview in the Methods section.

*Response: Thank you for your suggestion. We have revised the introduction to include brief information of the screening programmes in Denmark. We believe this will enhance the clarity of the manuscript.*

*“Participating in one screening programme increases the probability of participating in other screening programmes (7-9). While the participation rate in BC screening in Denmark has consistently remained high (currently above 80%) (10), the participation rates of both CC and CRC screening remain around 60% (10), resulting in a participation gap in these two screening programmes (9), despite all programmes being offered free of charge. The same picture is seen in other Western countries with organised screening programmes and comparable healthcare services (11-13).*

*In Denmark, screening invitations are sent directly to residents within the eligible age range whenever they are due. Women aged 50-69 years receive a prescheduled appointment for breast cancer screening, while women aged 23-64 are invited to book appointments for cervical cancer screening. Both women and men aged 50-74 receive self-sampling kits for colorectal cancer screening. This approach results in a cohort of women who are eligible for all three screening programmes at the same time.”*

21.	-It is correct that there are multiple reasons for non-participation. However, literature indicates that some populations may face unique barriers (e.g. language barriers, discrimination) that prevent them from engaging in screening. It would be beneficial to highlight any barriers specific to Denmark in this section.

*Response: To our knowledge, Denmark does not face unique barriers compared to other Western countries. In the discussion, we have elaborated on the barriers to non-participation at the individual, relational and healthcare levels. Danish healthcare is provided free of charge, therefore, economic constraints are not a major barrier. Additionally, the country’s small size and decentralised screening services, including multiple mammography clinics within each region, high accessibility to GPs and self-sampling offers in CRC screening, contribute to improved accessibility for screening services.*

Methods

Study Population

22.	-This study indicates that the study focused on women. Were individuals who identify as gender non-binary and transgender men with a cervix included in this study population? International studies indicate that these populations experience greater under screening that cis-gender women.

*Response: Thank you for your inquiry. The BC screening invitations are generated through an integrated call-recall system, which automatically sends invitations to individuals with CPR numbers (civil personal registration number) designated as female gender. These CPR numbers are assigned at birth or immigration, but individuals have the option to apply for a change in their CPR number in the event of a legal gender transition. If an individual with a male-designated CPR number is biologically female (assigned female at birth), they are eligible to apply for screening. We included all individuals attending BC screening during the intervention period, irrespective of their CPR number. However, we do not have information regarding their gender.*

1.	Rao A, Young S, Erlich H, Boyle S, Krevolin M, Sun R, et al. Development and characterization of the cobas human papillomavirus test. J Clin Microbiol. 2013;51(5):1478-84.

2.	Tranberg M, Jensen JS, Bech BH, Blaakaer J, Svanholm H, Andersen B. Good concordance of HPV detection between cervico-vaginal self-samples and general practitioner-collected samples using the Cobas 4800 HPV DNA test. BMC Infect Dis. 2018;18(1):348.

3.	Flowchart for women aged 23-64 years, self-sampled HPV screening [in Danish] Accessed April 2024: The National Steering Committee for Cervical Cancer Screening (NSLS) [Available from: <https://www.fagperson.regionshospitalet-randers.dk/globalassets/hospitaler/rh-randers/fagperson/afdelinger/afdeling-for-folkeundersogelser/nsls/dokumentsamling/flowchart-23-64-ar-selvopsamlede-prover.-nsls-godkendt-21.-september-2022.pdf>.

4.	Flowcharts for HPV screening [in Danish]. Accessed April 2024: The National Steering Committee for Cervical Cancer Screening (NSLS), 2020; [Available from: <https://www.fagperson.regionshospitalet-randers.dk/afdelinger/afdeling-for-folkeundersogelser/national-styregruppe-for-livmoderhalskraeftscreening-nsls/udgivelser-og-links-i-den-nationale-styregruppe-for-livmoderhalskraftscreening-nsls/>.

5.	Manual for implementering og drift af tværregional tarmkræftscreening [in Danish] Danske Regioner, 2017.

6.	van Baars R, Bosgraaf RP, ter Harmsel BW, Melchers WJ, Quint WG, Bekkers RL. Dry storage and transport of a cervicovaginal self-sample by use of the Evalyn Brush, providing reliable human papillomavirus detection combined with comfort for women. J Clin Microbiol. 2012;50(12):3937-43.

7.	Labeit A, Peinemann F. Breast and cervical cancer screening in Great Britain: Dynamic interrelated processes. Health Econ Rev. 2015;5(1):32.

8.	Poiseuil M, Moutel G, Cosson M, Quertier MC, Duchange N, Darquy S. Participation in breast cancer screening and its influence on other cancer screening invitations: study in women aged 56 years old in four French departments. Eur J Cancer Prev. 2023.

9.	Njor SH, Søborg B, Tranberg M, Rebolj M. Concurrent participation in breast, cervical, and colorectal cancer screening programmes in Denmark: A nationwide registry-based study. Prev Med. 2023;167:107405.

10.	Sundhedsstyrelsen. Status report on the national cancer screening programmes [Statusrapport for de nationale screeningsprogrammer for kræft, by the Danish National Health Authority]. <https://sst.dk/da/udgivelser/2022/statusrapport-for-de-nationale-screeningsprogrammer-for-kraeft>; 2022.

11.	Kregting LM, Olthof EMG, Breekveldt ECH, Aitken CA, Heijnsdijk EAM, Toes-Zoutendijk E, et al. Concurrent participation in breast, cervical, and colorectal cancer screening in the Netherlands. Eur J Cancer. 2022;175:180-6.

12.	Rebolj M, Parmar D, Maroni R, Blyuss O, Duffy SW. Concurrent participation in screening for cervical, breast, and bowel cancer in England. J Med Screen. 2020;27(1):9-17.

13.	Dawidowicz S, Le Breton J, Moscova L, Renard V, Bercier S, Brixi Z, et al. Predictive factors for non-participation or partial participation in breast, cervical and colorectal cancer screening programmesdagger. Fam Pract. 2020;37(1):15-24.
