# Supplementary material for: Increasing coverage in cervical and colorectal cancer screening by leveraging attendance at breast cancer screening: A cluster-randomised, crossover trial
Source: PLoS Med. 2024 Aug 13;21(8):e1004431. doi: 10.1371/journal.pmed.1004431 (PMC11321549; doi:10.1371/journal.pmed.1004431)
Supplement: Attachment — Submitted filename: Response to editorial comments_070624.docx [file pmed.1004431.s008.docx]

Dear Alexandra Tosun

Thank you for affording us the opportunity to revise our manuscript once more.

Below, you’ll find our comprehensive response to the editorial comments, presented point by point with our answers in italics. We have diligently addressed each comment and are grateful for the valuable feedback provided by the editors.

We trust that our revisions meet your expectations. Should you have any clarifying questions, please do not hesitate to reach out. We look forward to hearing from you.

Yours sincerely,

Anne Dorte Lerche Helgestad

*On behalf of all authors*

GENERAL COMMENTS

We encourage you to include the original protocol document and analysis plan, with any amendments, as Supporting Information to be published with the manuscript if accepted (along with the published version of the protocol).

*Thank you for your inquiry. However, the published version of the protocol and analysis plan is the only document we have adhered to, and no additional protocol document exists.*

Under data availability you have stated that “The participants were not asked to give consent for publication of the questionnaire data.” whereas in lines 296-298, you have stated that informed consent was not required. Please comment and update the details with appropriate information.

*We acknowledge that the differences can seem confusing. In the ethics statement, the indication that no informed consent is required refers to the inclusion of participants in the study. However, in the data availability statement, sharing the questionnaire data with external parties would require informed consent from the respondents, which was not obtained.*

*We have revised the ethics statement ll. 304-307 to provide clarity:*

*“In pursuance of the Danish Consolidation Act on Research Ethics Review of Health Research Projects, the study was not notifiable to the Committee (R. No.: 1-10-72-1-21) and informed consent was therefore not required for the register data used in the study.”*

Throughout the manuscript, please provide more detailed references to the results in the Supplementary Material (e.g. “S1 Table 2”).

We appreciate your response to the comment by Reviewer #4 regarding the inclusion of individuals who identify as gender non-binary and transgender men with a cervix. We encourage you to include these details in the Methods section and also in the discussion as appropriate.

*Thank you for your inquiry. We have revised with the following in the method section, ll. 156-157, and in the discussion, ll. 520-522:*

*“Individuals attending BC screening during the intervention period were included irrespective of their gender.”*

*“Additionally, we lack information on whether any of the targeted residents were transgender. Addressing inequities, including those among socioeconomic groups and between genders, may require supplementary interventions for better targeting.”*

ABSTRACT

1) l.36: Please describe briefly what “control” entails, for example, as done in lines 192-193.

*We have added the following to ll. 38-39:*

*“Women in the control group received only standard screening offers according to the organised programmes.”*

2) l.38: Please change to: “…and women with overdue screening were offered self-sampling.” or similar (please do not use the term “overdue women”). Please amend throughout the abstract and the main text.

*We have adjusted the terminology accordingly throughout the manuscript.*

3) l.42: Please introduce the abbreviation for confidence intervals here (“…with 95% confidence intervals (CI).”)

*We have included the abbreviation accordingly.*

4) l.54: Please remove the word “remarkable”.

*We have removed the word.*

5) l.56: Please correct the trial registration number to “NCT05022511”.

*We have corrected the registration number.*

AUTHOR SUMMARY

l.75: Please change to: “We conducted…”

*We have adjusted the sentence accordingly.*

INTRODUCTION

1) l.109: Please change “currently” to an exact time detail, e.g. “as of 2024”.

*We have revised the sentence as follows and included the most recent annual report as a reference:*

*“While the participation rate in BC screening in Denmark has consistently remained high (above 80% as of 2023) [19]…”*

2) l.115: When stating age, please add the relevant unit, e.g. “aged 23-64 years”. Please revise throughout the main text.

*We have revised the manuscript accordingly.*

METHODS AND RESULTS

1) l.161: “The rationale and design of this trial have been described in details elsewhere [25].”

*It appears that there might be something missing in the comment. Please inform me if any revisions are needed to clarify the sentence.*

2) l.212 “Outcomes”: The protocol lists the outcomes of [incidence of HPV-positive cases in women 60–64 years after 12 months with an initial negative HPV sample]. (a) Can you please present those results as part of this manuscript, or indicate why that is not possible? (b) If you do not wish to include these results in the current paper, can you please indicate when you plan to publish these results?

*Thank you for the inquiry. We have now been able to calculate this outcome, having sufficient follow-up time. It has been added to the methods, results and discussion sections as follows:*

*Ll. 232-233: “In CC screening, the response rate to the re-test offer and the incidence of HPV-positive cases after 12 months were calculated for women aged 60-64 years with an initial HPV-negative self-sample.”*

*Ll. 388-391: “Of the women aged 60-64 years with an initial negative HPV self-sample, a total of 75.6% (n/N=34/45, 95% CI [60.4, 87.1]) completed the self-sample re-test sent 12 months later. All women who complete the re-test were HPV negative.”*

*Ll. 464-467: “All women aged 60-64 years who performed HPV re-testing 12 months after participating in the intervention with an initial HPV-negative self-sample also tested negative for HPV in the re-test. This suggests that these women may potentially be eligible to exit the screening programme after a single HPV-negative self-sample.”*

3) ll.231-233: The following outcomes measures, “Secondary outcomes also included the proportion of women eligible for all three programmes, the proportion of these women overdue for both CC and CRC screening, and their participation in both screenings within the follow-up period.”, appear to differ between the submitted manuscript and the protocol. Please clarify and explain the discrepancy. If the outcomes were not prespecified in the protocol, please indicate that they were post hoc and explain why they were added. Post hoc comparisons should be presented as hypothesis generating rather than conclusive.

*Thank you for the important comment. We acknowledge the discrepancy between the outcome measures presented in the submitted manuscript and those outlined in the protocol. The decision to include additional outcomes was made post protocol to investigate the patterns among individuals eligible for all three programmes but who only participated in BC screening. However, we understand the importance of presenting such post hoc comparisons cautiously and without conclusions.*

*In response to your feedback, we have revised the method section with the following, ll. 237-240:*

*“Post-protocol, secondary outcomes also included the proportion of women eligible for all three programmes, the proportion of these women overdue for both CC and CRC screening, and their participation in both screenings within the follow-up period to describe participation patterns for women eligible in all three programmes and overdue for both CC and CRC screening.”*

4) Figure 2: Please add a footnote for the asterisk.

*A footnote has been added.*

5) Table 2: Regarding missing data, you stated that only descriptive data for socio-demographic variables were missing for a very small proportion of the study population. We understand that there may be limitations in presenting the data due to Danish data protection legislation. However, we wonder whether adding a row to report 'missing data' for each variable in Table 2 will identify a person. In any case, we have noticed that the calculated percentages are incorrect, as it appears that you have used denominators for the calculations that exclude those with missing data. Please revise and use the total N to calculate the percentages (which may then not equal 100%).

*The legislation does not permit us to present numbers lower than 3, even though it is unlikely to serve as an identifier. We have now included a row reporting the missing data but have masked the exact number in the two cases where the count is very low. The percentages have been recalculated using the total as the denominator, and following information has been added as a footnote:*

*“Exact numbers are not given in pursuance of Danish data protection legislation.”*

6) Table 2: Please add a definition for “Western immigrants” and “Non-western immigrants” and exchange the word “immigrant” with “migrant”. Also, please define “FIT” at first use in the footnote.

*We have revised the table text accordingly:*

*“Western immigrants: individuals originating from countries within the European Union, Andorra, Australia, Canada, Iceland, Liechtenstein, Monaco, New Zealand, Norway, San Marino, Switzerland and the USA. Non-Western migrants: originating from all other countries*.

*Further, we have defined FIT at first use in the footnote.*

7) Table 3: Please change “[82.9%, 84.1%9” to “[82.9%, 84.1%]” (i.e., missing end bracket).

*We have now included the missing bracket*.

8) Table 4: Please define “FIT” at first use in the footnote.

*We have now defined FIT in the footnote.*

9) l.371 ff: Please add a denominator when presenting results (e.g. “..was 8.0% (n/N=13/163; 95% CI [4.3%, 13.3%])”). Please revise throughout the main text.

*We have revised the presentation of the results including the denominator throughout the text.*

10) ll.375-377: Could you please comment on the restrictions based on Danish data protection legislation? It seems that in some cases it is okay to provide small numbers, and in others not. Please check if it is possible to provide an exact number, as the number alone is unlikely to be a personal identifier.

*Thank you for your comment. As detailed in comment 5), we are unable to present numbers lower than 3 due to regulations, even though they are unlikely to serve as personal identifiers. We have revised the manuscript to ensure coherence regarding this issue.*

11) ll.383 ff: Please present either the n number (along with a denominator) first, followed by the percentage in parentheses, or the other way around. Please avoid mixing. For example: “For CC screening, 81.3% (n=3,134) of the eligible women gave consent to the administrative check up on their CC screening status, with 5.3% (n=204) declining.”

*We have revised the text for consistency in the presentation.*

12) l.398: Please report the exact p-value.

*We have included the exact p-value now.*

13) Table 5: Please include the denominators for the different groups and check that the percentages were calculated appropriately. “Women indicating that this was not relevant for them were excluded.” – We feel that all respondents should be included; please explain why these women were excluded.

*Thank you for your comment. We acknowledge that this can be unclear. The questionnaire was sent to all women attending BC screening, and the responses were evaluated collectively for women eligible for CC or CRC screening, as well as those eligible for both. Women had the option to indicate that a similar intervention would not be relevant for them if they were no longer within the screening-eligible age range at the time of the next invitation or if they had a history of cancer or hysterectomy.*

*We have elaborated this in ll. 445-447, and included denominators for the different groups in the table:*

*“Women indicating that this was not relevant for them were excluded (if they were no longer within the screening-eligible age range at the time of the next invitation or if they had a history of cancer or hysterectomy).”*

DISCUSSION

1) We feel that the discussion is currently a bit disjointed. For example, the limitations of the study are not presented in a dedicated paragraph but are interspersed between other paragraphs. We would encourage you to revise the discussion to improve clarity and structure; typically, limitations are discussed in a standalone paragraph.

*Thank you for addressing this matter. We fully agree that revising and tightening up the discussion for clarity and structure would enhance its quality. We have thoroughly revised the discussion, ensuring that none of the key points have been omitted, and we have now consolidated limitations into one section. We trust that this revision will meet your expectations.*

2) ll.428-429/l.514: Please temper claims of primacy of results by stating, "to our knowledge" or something similar.

*We have tempered the primacy ll. 457-458 with the following:*

*“To our knowledge, this is the first study directly using high coverage in one cancer screening programme to increase participation in others, and this was successful.”*

3) ll.437-443: We feel that the message this paragraph is intended to convey is currently unclear. Please revise for clarity.

*We have revised the paragraph, ll. 468-474, in an effort to clarify our point. We hope this will meet your expectations.*

4) l.462: “This bodes well for the generalisability of the study findings” – We feel that this statement requires a bit more context, as the results of the study could be generalized to Denmark as a whole or to countries with similar health care systems.

*We have revised the sentence for context (ll. 488-489):*

“This bodes well for the generalisability of the study findings to other Danish regions and to countries with comparable healthcare systems.”

5) l.527: Please remove the “Conclusion” subheading.

*We have removed the subheading.*

SUPPLEMENTARY MATERIAL

Thank you for providing the CONSORT checklist. Please replace the page numbers with paragraph numbers per section (e.g. "Methods, paragraph 1"), since the page numbers of the final published paper may be different from the page numbers in the current manuscript. Also, please add the following statement, or similar, to the Methods: "This study is reported as per the Consolidated Standards of Reporting Trials (CONSORT) guideline for cluster randomised trials (S1 Checklist)."

*We have included the following statement within the method section, ll. 173-174:*

*“The study is reported as per the Consolidated Standards of Reporting Trials (CONSORT) guideline for randomised trials (S2 CONSORT Reporting checklist).”*

REFERENCES

Where website addresses are cited, please specify the date of access including day, month and year (e.g. [accessed: 16/09/2023]).

*We have revised the reference list accordingly.*

SOCIAL MEDIA

To help us extend the reach of your research, please provide any X (formerly known as Twitter) handle(s) that would be appropriate to tag, including your own, your co-authors’, your institution, funder, or lab. Please enter in the submission form any handles you wish to be included when we post about this paper.

*Thank you for the inquiry. Unfortunately, we do not have an account on X. However, we appreciate tour efforts to extend the reach of our research.*

Comments from Reviewers:

Reviewer #3: Thank you to the authors for addressing all my previous comments well. I have no further issues to raise.
