# Supplementary material for: Increasing coverage in cervical and colorectal cancer screening by leveraging attendance at breast cancer screening: A cluster-randomised, crossover trial
Source: PLoS Med. 2024 Aug 13;21(8):e1004431. doi: 10.1371/journal.pmed.1004431 (PMC11321549; doi:10.1371/journal.pmed.1004431)
Supplement: Attachment — Submitted filename: Response to editorial comments_140624.docx [file pmed.1004431.s009.docx]

Dear Alexandra Tosun,

Below, you will find the revisions in response to the editorial comments, presented point by point with our responses indicated in italics.

We trust that our revisions align with your expectations. However, please feel free to contact us if further clarifications or revisions are required.

Kind regards,

Anne Dorte Lerche Helgestad

*On behalf of all the authors*

Requests from Editors:

1) COMPETING INTERESTS STATEMENT

please replace ‘proces’ with ‘process’.

*We have corrected the word.*

2) ABSTRACT

In the last sentence of the methods and findings section please briefly detail the main limitations of the study’s methodology.

*We have included the following sentence:*

*“Women who did not participate in BC screening were not included in this study.“*

3) AUTHOR SUMMARY

Please swap bullet points at lines 83 and 85 to ensure bullet point at line 85 is the first bullet point. Please also revise line 85 to read, ‘This study suggests that combining preventive screening services…’

Line 86 – it isn't entirely clear what i meant by 'health offers'. Suggest, ‘…public health interventions…’ instead perhaps?

Line 87 – suggest, ‘…and is likely to require a distinct approach…’

In the final bullet point of the ‘What do these findings mean sub-section’ please include the main limitations of the study in non-technical language.

*The lines have been revised for readability as follows:*

*“This study suggests that combining preventive services may offer a pragmatic strategy to enhancing the effectiveness of public health interventions in various settings.”*

*In the final bullet, we have revised the sentence for readability as suggested and to explicitly state that this is our main limitation:*

*“The main limitation of the study is that the intervention focuses solely on women attending BC screening, and a different approach is required for women not participating in this screening.”*

4) METHODS AND RESULTS

Line 161: “The rationale and design of this trial have been described in details elsewhere [25].” Please replace ‘details’ with ‘detail’.

*It has been corrected.*

5) TABLE 2 FOOTNOTE

For explicit clarity suggest, “Exact numbers are not given in pursuance of Danish data protection legislation, which does not permit reporting of individual data where total numbers are lower than 3.”

Line 330 – please revise ‘**Western immigrants’ to read ‘**Western migrants’. Please check carefully for the same elsewhere and amend throughout all subsections of the main manuscript and supporting files as necessary.

*We have changed the footnotes accordingly and carefully applied the revisions throughout the text.*

6) TABLE 5

Line numbers are overwritten on the final column of the table, please amend.

*We have now amended the line numbers*

7) STATEMENTS OF DECLARATION

Lines 560 and 567 – please remove these statements from the end of the discussion and include only in the manuscript submission form. They will be compiled as metadata at the time of publication.

*They have now been removed.*

8) SUPPORTING INFORMATION

Please label ‘S1 File supplementary material’ as ‘S1 Appendix’.

Within the S1 Appendix please label tables as ‘S1 Table, S2 Table’ etc in numbered sequence and similarly, figures as ‘S1 Fig, S2 Fig’.

Please label ‘S2 File CONSORT’ as ‘S2 CONSORT Checklist’.

Please be reminded to amend citations to the supporting information files/figures/tables throughout all sections of the manuscript (and supporting files).

Please be reminded to amend the titles/captions at line 699 onwards.

*We have revised the labeling of the supporting information.*

9) STUDY PROTOCOL

There seems to be some confusion regarding the required study protocol. A reference and a citation to the published BMJ article will suffice, we do not need to re-publish this item.

Please remove the PDF file of the BMJ article and instead upload a copy of the protocol document which would have been submitted as part of the trial approval process. The BMJ article was published sometime after the trial commenced thus cannot be the ‘original’ protocol (it is the published version of the original protocol).

Please label the uploaded protocol document as ‘S3 Protocol’.

*We have not removed the published protocol from the PDF file.*

*As stated under 'Ethics Statement', this study was not deemed to be notifiable to the Ethical Committee. Additionally, listing with the Central Denmark Region does not require a protocol. This is because the intervention is considered a reminder of an already existing healthcare offer for the women in the target group. Therefore, we cannot upload any protocol other than the BMJ protocol, as no other protocol exist.*
